# Acute Effects of a Low Dose of Caffeine on Physiological and Performance Factors in Young Male Soccer Players: A Randomized Crossover Study

**DOI:** 10.1016/j.cdnut.2026.109528

**Published:** 2026-09-03

**Authors:** Mohammad Mehdi Azizi, Mohsen Salesi

**Affiliations:** Department of Sport Sciences, Shiraz University, Shiraz, Iran

**Keywords:** heart rate, blood pressure, rate pressure product, Loughborough soccer passing test, Yo-Yo intermittent recovery test level 1

## Abstract

**Objectives:**

This research aimed to study the acute consumption of caffeine on the performance of young male soccer players in the Loughborough soccer passing test (LSPT) and Yo-Yo intermittent recovery test level 1 (YYIR1), and also on the heart rate (HR), blood pressure (BP), and rate pressure product (RPP), immediately after the YYIR1.

**Methods:**

In a randomized, counterbalanced, crossover design, 12 young male soccer players (16.5 ± 0.52 y; 63 ± 8.06 kg; training background: 4–5 sessions/wk) voluntarily completed 2 distinct experimental sessions: *1*) caffeine and *2*) placebo. In each test session, 1 h after taking a caffeine or placebo (flour) capsule (3 mg·kg^-1^), the participants first completed the LSPT and then the YYIR1. HR, systolic BP (SBP), and diastolic BP (DBP) were measured before the start, 1 h after the start, and immediately after the end of the YYIR1. Because the Shapiro–Wilk test confirmed the normality of the data, parametric tests [repeated-measures analysis of variance (ANOVA) and dependent *t*-test] were used to analyze the research questions.

**Results:**

Two-way repeated-measures ANOVA revealed significant main effects of condition (*P* = 0.006) and time (*P* < 0.001) for HR, without a significant condition × time interaction (*P* = 0.144); however, post hoc analysis showed significantly higher HR after caffeine ingestion compared with placebo immediately after the YYIR1 (*P* = 0.025). A significant condition × time interaction was observed for DBP (*P* = 0.014), although pairwise comparisons showed no significant post hoc differences (all *P* ≥ 0.101). No significant main or interaction effects were found for SBP or RPP (all *P* ≥ 0.066).

**Conclusions:**

Within the limits of the current sample size, acute ingestion of 3 mg·kg^-1^ caffeine elicited modest physiological responses in adolescent soccer players, significantly augmenting postexercise HR without substantially altering BP, RPP, or soccer-specific performance factors. However, given the observed numerical improvements in functional test results, these findings should be interpreted cautiously, as low-dose caffeine may offer subtle practical benefits that warrant further investigation with greater statistical power.

## Introduction

The utilization of nutritional supplements and ergogenic aids is a widespread practice among elite competitors aiming to maximize athletic success. Within this context, caffeine represents one of the most frequently administered stimulants, owing to its proven capability to boost physical capacity in both individual disciplines and team-based sports [1].

Indeed, it has been reported that ∼74% of elite athletes use caffeine as an ergogenic aid before or during competition [2]. Because many team sports, such as basketball, hockey, and soccer, utilize repeated bouts of short sprints separated by active recovery periods, interval running may be applicable to many sports and sensitive to nutritional supplements that are designed to delay high-intensity, exercise-induced fatigue [3]. Caffeine supplementation is beneficial for high-intensity activities, including team sports such as soccer and rugby, which are defined by intermittent efforts performed over prolonged durations [4].

Soccer performance relies on a combination of continuous endurance exercise and repeated sprint actions. Official match play consists of two 45-min halves separated by ∼15 min of rest, with total match duration potentially extending to 120 min if extra time is played. The number of sprint actions and the total distance covered at sprinting speeds are key performance indicators that differentiate elite from subelite soccer players. Athletes who are capable of sprinting more frequently and over longer distances during match play are generally more likely to compete at higher competitive levels [5]. Caffeine has been consistently reported to exert positive effects on physical performance in endurance-based activities, intermittent exercise approaches such as team and racket sports, and high-intensity events lasting between 1 and 60 min, including swimming, rowing, and middle- to long-distance running. Notably, the ergogenic properties of caffeine during exercise have been recognized in the scientific literature for >1 century [6]**.**

At the mechanistic level, caffeine is structurally similar to adenosine and acts as a nonselective antagonist of adenosine receptors (A1, A2a, and A2b). Through this mechanism, it functions as a potent central nervous system stimulant and enhances the synthesis of catecholamines with neurotransmitter activity, such as dopamine, epinephrine, and norepinephrine. Given the abundance of adenosine receptors in skeletal muscle, caffeine ingestion can increase neuromuscular recruitment. Moreover, at the muscular level, caffeine increases the frequency of calcium channel opening, thereby facilitating calcium release into the myoplasm and augmenting muscle contraction [7]. The ergogenic efficacy of caffeine is influenced by 3 primary factors: dosage and timing of intake, the type of sport or exercise performed, and individual responsiveness to caffeine. Importantly, there is no linear dose–response relationship between caffeine intake and the magnitude of its performance-enhancing effects [8]. Variability across studies, including differences in exercise protocols, participant characteristics (e.g., age, sex, training status, and experience), caffeine dose, and genetic predisposition, may account for inconsistencies in reported outcomes regarding caffeine’s ergogenic action [9]. Previous research has demonstrated that ingestion of 6 mg·kg^-1^ of caffeine 1 h before testing significantly improved reaction time in soccer players. Other findings suggest that caffeine consumption may enhance athletic performance and soccer-specific skills [10].

In a study examining sprint performance during the Loughborough intermittent shuttle test, administration of 3.7 mg·kg^-1^ of caffeine to male soccer players resulted in significant reductions in sprint times during the final 3 stages of the protocol [11]. Similarly, investigating the acute effects of caffeine intake on muscular strength and endurance in male amateur soccer players revealed that a dose of 5 mg·kg^-1^ significantly improved both outcomes [12]. Additional research involving professional soccer players assessed the effects of caffeine supplementation (6 mg·kg^-1^) on Hoff and sprint performance tests. The findings revealed significant improvements in Hoff test mean scores and statistically significant reductions in sprint times compared with both placebo and nonsupplementation conditions. It was reported that female soccer players who consumed 3 mg·kg^-1^ of caffeine exhibited significantly greater jump performance and faster sprint times than placebo conditions. Furthermore, caffeine supplementation has been reported to positively influence test duration in soccer players, likely because of enhanced nervous system stimulation that promotes increased alertness and physical readiness [13].

Although higher doses of caffeine (6–9 mg·kg^-1^) have been traditionally investigated, evaluating a lower dose of 3 mg·kg^-1^ is of particular practical relevance for adolescent athletes. This specific dosage is commonly chosen to balance potential ergogenic benefits with the minimization of adverse side effects, such as increased anxiety, sleep disturbances, or excessive cardiovascular strain, which are more pronounced in younger populations.

Despite the growing body of evidence, limited research has simultaneously examined the effects of caffeine on both physiological responses [heart rate (HR), blood pressure (BP), rate pressure product (RPP)] and soccer-specific performance tests [Loughborough soccer passing test (LSPT) and Yo-Yo intermittent recovery test level 1 (YYIR1)] in young athletes. Therefore, the primary objective of this study was to investigate the acute effects of caffeine supplementation at 3 mg·kg^-1^ on these outcomes in young male soccer players. We hypothesized that caffeine ingestion would *1*) improve LSPT performance and YYIR1 distance and estimated maximal oxygen consumption (VO_2max_) compared with placebo, and *2*) increase HR, systolic BP (SBP), and RPP after the YYIR1, reflecting a greater cardiovascular response to exercise under caffeine conditions.

## Methods

### Participants

Twelve healthy young males (16.5 ± 0.52 y old) with ∼3 y of advanced soccer training experience and participation in ≥4 training sessions per week volunteered to take part in this study. All participants were determined to be postpubertal (Tanner stage 5) based on self-assessment documentation and physical characteristics, ensuring a homogeneous physical maturation status. The participants’ anthropometric characteristics are summarized in Table 1. To ensure compliance with caffeine restrictions, participants were provided with a comprehensive list of common caffeine-containing foods and beverages and were instructed to avoid these items for the duration of the study. All participants refrained from the use of dietary supplements, medications, tobacco, alcohol, and caffeinated products for 72 h before the initiation of data collection and throughout the experimental period.

**TABLE 1:** Characteristics of participants (*n* = 12)

Characteristic	Mean ± SD
Age (y)	16.5 ± 0.52
Height (cm)	172 ± 8
Weight (kg)	63 ± 8
Daily caffeine intake (mg·d^-1^)	64 ± 15

Furthermore, a 3-d dietary recall was obtained before each intervention session to verify adherence to dietary guidelines and to confirm the absence of inadvertent caffeine intake. Habitual caffeine consumption was assessed using the Center for Science in the Public Interest database (www.cspinet.org; accessed July 1997) [14], indicating that all participants were classified as low-caffeine consumers (64 ± 15 mg·d^-1^). This study was conducted in accordance with the Declaration of Helsinki and was approved by the Ethics Committee of Islamic Azad University, Kazerun Branch (approval code: IR.IAU.KAU.REC.1402.039). To ensure a true crossover design and eliminate order effects, participants were assigned to their initial condition using a block randomization sequence generated by an independent researcher. A double-blind protocol was strictly maintained; both the caffeine and placebo capsules were identical in appearance, color, size, and weight, and were distributed in coded containers to ensure complete concealment of the sequence from both the participants and the primary investigators until all statistical analyses were completed. All participants resided in the same training camp and followed an identical, supervised training program. They were also instructed to avoid strenuous physical activity for 48 h before each testing session.

### Sample size and study design

The required sample size was determined a priori using G∗Power software (Heinrich Heine University Düsseldorf, Düsseldorf, Germany) [15] for a 2-tailed paired *t*-test within a 2-period crossover design. The calculation was specifically centered on the total completion time in the LSPT as the primary outcome variable. Assuming a type I error rate of 5% (α=0.05), a statistical power of 80% (1−β=0.80), an expected effect size of d=0.224 for functional time improvements over placebo [16], and a high within-subject correlation (r=0.90) characteristic of repeated measures in crossover designs, a minimum sample size of 12 participants was calculated to be sufficient. Secondary outcome variables included YYIR1 performance metrics [covered distance (CD) and estimated ${\text{VO}}_{2\;\max}$] and postexercise cardiovascular responses (HR, BP, and RPP).

Investigators responsible for performance testing and data analysis were blinded to the experimental conditions to reduce potential bias. Before study initiation, participants attended a familiarization session during which all testing procedures and protocols were explained. Baseline measurements of age, height, and body mass were obtained. Participants also completed *1*) a written informed consent form confirming their understanding of the study procedures, potential benefits, risks, and possible adverse effects; and *2*) a questionnaire assessing caffeine intake and intolerance. A randomized, counterbalanced, double-blind, crossover design was implemented. All 12 participants completed both experimental conditions (caffeine and placebo), separated by a 1-wk washout period to eliminate any carryover effects. Half of the participants randomly initiated the first session with caffeine, whereas the remaining half started with the placebo, reversing the order in the subsequent session. Given that the metabolic half-life of caffeine is ∼3 to 7 h, this 1-wk washout interval was more than sufficient to ensure complete clearance. Furthermore, exploratory analysis confirmed the absence of any significant period or sequence effects (P>0.05) between the 2 testing sessions.

Supplements were administered according to the specified protocol, after which participants completed the LSPT, followed by the YYIR1. HR and BP were recorded before supplementation (pre), 60-min postsupplementation (post), and immediately after completion of the YYIR1 (post-YYIR1).

### Caffeine supplementation and placebo

In the caffeine condition, PARTICIPANTS ingested pure anhydrous caffeine (cat. no. C0750; Sigma-Aldrich) at a dose of 3 mg·kg^-1^ body mass with 200 mL of water, 60 min before testing. In the placebo condition, participants consumed an identical capsule containing flour 60 min before the tests. Participants consumed a breakfast providing 250 kcal (45 g carbohydrate, 9 g protein, and 5 g fat) 90 min before each testing session, and all exercise trials were conducted at the same time of day (09:00–13:00). Water intake was restricted during testing to minimize interparticipant variability in gastrointestinal content and to prevent potential dilutional effects on physiological measurements. This restriction was applied equally across both conditions (caffeine and placebo) to ensure consistency.

Environmental conditions within the testing facility were kept consistent across sessions (temperature: 21–23°C; relative humidity: 50%–55%). Participants were instructed to maintain their habitual sleep pattern (minimum 7 h/night) and to refrain from staying up late the night before each testing session. Sleep quality, duration, and compliance with dietary and lifestyle restrictions were verified via self-report questionnaires on the morning of each session. Hydration status was standardized by instructing participants to consume 500 mL of water the night before and an additional 350 mL 2 h before arrival, which was verbally confirmed upon arrival at the laboratory.

### LSPT

The LSPT was conducted within a 12 × 9.5-m grid (Figure 1). Four gymnasium benches served as target areas, equipped with colored cards and aluminum strips to provide audible feedback upon successful hits. Participants performed passing sequences within the designated zones marked by cones and tape.

**FIGURE 1:**
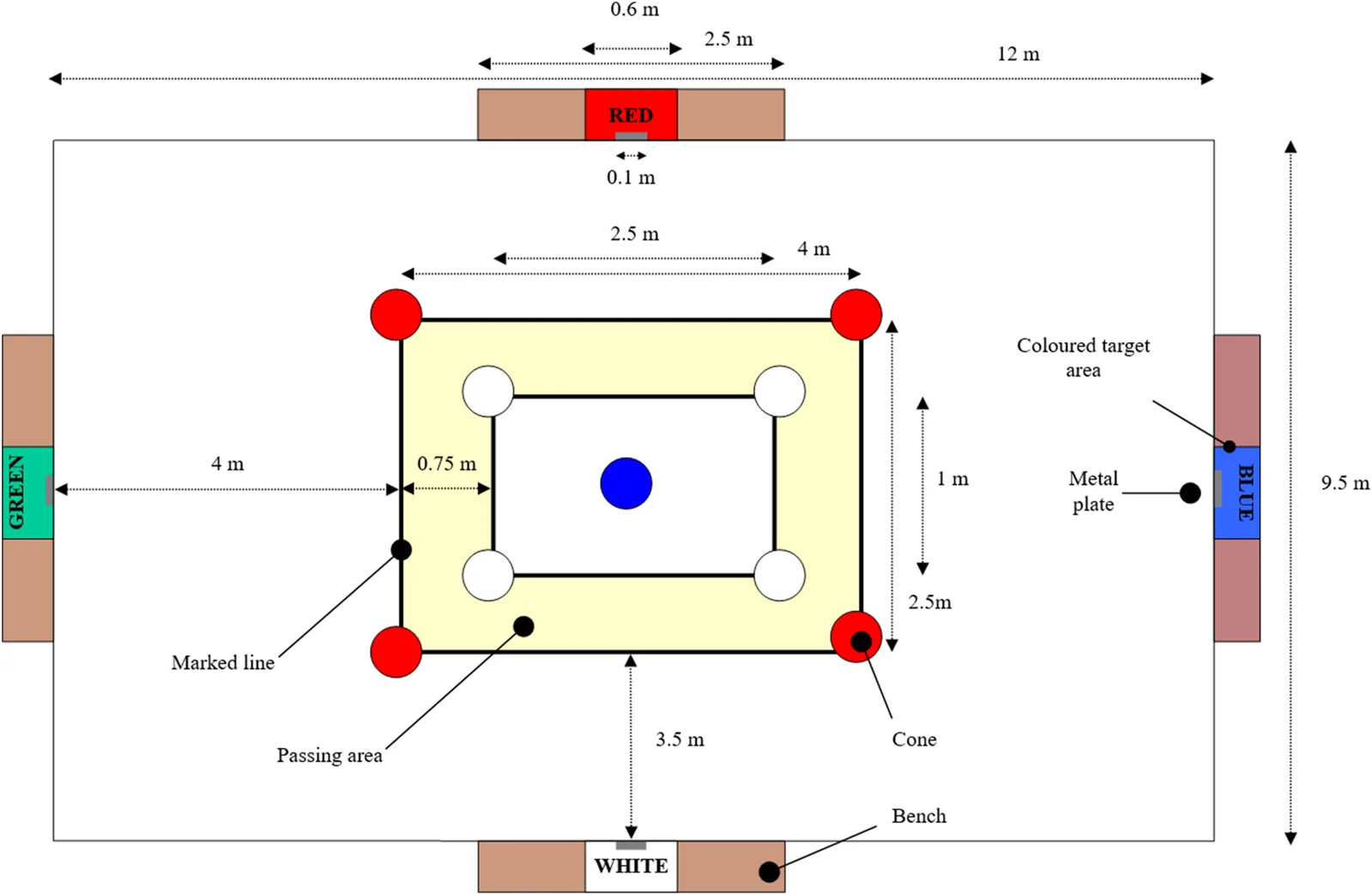
Schematic representation of the Loughborough soccer passing test.

The evaluation commenced with the soccer ball positioned adjacent to the central cone. At the instant the soccer player made initial contact to move the ball out of the inner rectangular zone, the primary examiner initiated a hand-held stopwatch. Concurrently, the secondary examiner was responsible for audibly indicating the subsequent target color sequence. This directional cue was provided immediately preceding the completion of the ongoing pass. The secondary examiner also stopped the clock when the last pass made contact with the target area and recorded all penalty time points during the trials. To minimize intertester variability, examiners maintained identical roles throughout all testing sessions. The specific sequence of passes followed 1 of 4 randomized configurations pregenerated by the research team, ensuring that each complete trial encompassed a balanced distribution of 8 long passes (targeting green and blue zones) and 8 short passes (targeting white and red zones). Participants were informed that passes must be executed strictly from within the passing area, specifically between the set of marked lines (as illustrated in Figure 1), and that upon retrieval from the previous pass, the ball had to cross 2 of the inner marked lines before the next attempt could be made. No feedback regarding performance times or errors was provided during the sessions.

The monitoring examiner positioned themselves to maintain an unobstructed view of all 4 target benches. Standardized error penalties were calculated and added to the total time as follows:

• A 5-s penalty was accumulated if a pass completely missed the designated bench or was directed to an incorrect target.
• A 3-s penalty was recorded whenever the ball failed to hit the specific (0.6×0.3 m) target zone.
• A 3-s penalty was assigned for any illegal handling of the ball.
• A 2-s penalty was given if the participant executed a pass from outside the boundaries of the passing sector.
• A 2-s penalty was added every time the ball made contact with any marking cone.
• A 1-s penalty was appended for each second exceeding the default 43-s completion threshold.
• Conversely, a 1-s deduction was credited to the overall score if the ball successfully struck the 10-cm central strip [17].

### YYIR1

The YYIR1 consisted of 20-m shuttle runs back and forth between 2 lines, with a gradual incremental increase in speed over time. A jogging distance of 5-m recovery zone was set behind the start line, with participants given 10-s resting recovery after each bout of shuttle running. The running speed was controlled by digital audio bleeps from a laptop.

The running speed of the first 4 bouts (0–160 m) was set at 10 to 13 $\text{km}\cdot h^{-1}$, and the subsequent 7 bouts (160–440 m) were 13.5 to 14 $\text{km}\cdot h^{-1}$. After the 11th bout, the running speed increased by increments of 0.5 $\text{km}\cdot h^{-1}$ every 8 bouts (i.e., after 760 m, 1080 m, 1400 m, 1720 m, etc.). The CD and maximum oxygen consumption (VO_2max_) were recorded when the participants failed to return to the start line a second time (a verbal warning was given to the participants in the first instance that they failed to make the line in time).

The validity of the YYIR1 to assess aerobic capacity has previously been described, with test-retest reliability between 0.78 and 0.98. Each participant’s predicted VO_2max_ was calculated by the equation: distance in meters × 0.0084 + 36.4 [18].

### HR and BP

HR and BP [SBP and diastolic BP (DBP)] were measured simultaneously using an automated digital upper-arm BP monitor before supplementation, 60 min after supplementation, and immediately after the completion of the YYIR1.

### RPP

To determine the energy requirement and establish the amount of stress put on the heart during exercise, cardiac specialists or exercise physiologists use the RPP. It is an observation of myocardial oxygen consumption, representing the internal myocardial workload when the heart beats, whereas the external myocardial work is a reflection of different stages of exercise. Expressed as the product of SBP and HR, the RPP is calculated by multiplying the SBP by the HR and dividing the product by 100 [19] (i.e., RPP = SBP × HR/100).

### Statistical analysis

Data were analyzed using the Statistical Package for the Social Sciences (SPSS version 28 [IBM Corp., Armonk, NY, USA]). The normality of the data distribution was determined using the Shapiro–Wilk test, and Mauchly’s sphericity test was used to check the homogeneity of the variance-covariance. A 2-way repeated-measures analysis of variance [ANOVA; 2 conditions (caffeine compared with placebo) × 3 times (presupplementation, 60 min postsupplementation, and post-YYIR1)] was performed to evaluate the effects of the interventions on the physiological variables. Also, the Bonferroni post hoc test was used to determine pairwise differences. In addition, a dependent *t*-test was used to evaluate the effect of the interventions on the functional variables. The level of statistical significance was *P* ≤0.05. Figure production was also performed using GraphPad Prism version 9.0.0 (GraphPad Software, San Diego, CA, USA).

## Results

### Physiological factors

Two-way repeated-measures ANOVA revealed a significant main effect of time (*F*_2_,_22_ = 58.75, *P* < 0.001, $\eta_{p}^{2}$ = 0.842) and a significant main effect of condition (*F*_1_,_11_ = 11.81, *P* = 0.006, $\eta_{p}^{2}$ = 0.518) for the HR. However, the condition × time interaction was not significant (*F*_2_,_22_ = 2.12, *P* = 0.144, $\eta_{p}^{2}$ = 0.161). Bonferroni pairwise comparisons demonstrated that HR increased significantly from pre to post (*P* = 0.002) and from pre to post-YYIR1 (*P* = 0.001) under the placebo condition, whereas HR was also significantly higher at post-YYIR1 than at post (*P* = 0.004). Similarly, under the caffeine condition, HR was significantly higher post-YYIR1 compared with both pre (*P* < 0.001) and post (*P* < 0.001), whereas the increase from pre to post did not reach statistical significance (*P* = 0.064). Pairwise comparisons between conditions revealed no significant differences at pre (*P* = 0.128) or post (*P* = 0.796), whereas HR was significantly higher after caffeine than placebo after YYIR1 (*P* = 0.025).

The analysis showed a significant main effect of time (*F*_2_,_22_= 4.64, *P* = 0.038, $\eta_{p}^{2}$ = 0.297) for SBP, whereas neither the main effect of condition (*F*_1_,_11_ = 0.005, *P* = 0.945, $\eta_{p}^{2}$ < 0.001) nor the condition × time interaction (*F*_2_,_22_ = 0.47, *P* = 0.623, $\eta_{p}^{2}$ = 0.041) reached statistical significance. The post hoc test indicated that there were no significant differences among pre, post, and post-YYIR1 within either placebo (all *P* ≥ 0.084) or caffeine (all *P* ≥ 0.129) conditions. Likewise, no significant differences were observed between placebo and caffeine at pre (*P* = 0.475), post (*P* = 0.980), or post-YYIR1 (*P* = 0.744).

Two-way repeated-measures ANOVA revealed no significant main effect of time ($F_{2,22}=0.07,P=0.935,\eta_{p}^{2}=0.006$) or condition ($F_{1,11}=0.25,P=0.626,\eta_{p}^{2}=0.022$) for DBP. However, a significant condition × time interaction was observed ($F_{2,22}=5.25,P=0.014,\eta_{p}^{2}=0.323$), indicating distinct temporal trajectories in DBP between conditions. Nevertheless, after Bonferroni correction for multiple comparisons, no specific pairwise differences reached statistical significance between conditions at pre (P=0.071), post (P=0.101), or post-YYIR1 (P=0.156), nor across time points within either condition (all P≥0.142). This discrepancy is likely attributable to the conservative nature of the Bonferroni adjustment in a small sample size (n=12).

The statistical analysis revealed a significant main effect of time ($F_{2,22}$= 44.83, *P* < 0.001, $\eta_{p}^{2}$ = 0.803), whereas neither the main effect of condition ($F_{1,11}$ = 4.17, *P* = 0.066, $\eta_{p}^{2}$ = 0.275) nor the condition × time interaction ($F_{2,22}$ = 1.96, *P* = 0.180, $\eta_{p}^{2}$ = 0.151) was significant for RPP. The post hoc test indicated that RPP increased significantly from pre to post (*P* = 0.045), from pre to post-YYIR1 (*P* = 0.001), and from post to post-YYIR1 (*P* = 0.004) under the placebo condition. Under the caffeine condition, RPP was significantly higher post-YYIR1 than both pre (*P* < 0.001) and post (*P* < 0.001), whereas the difference between pre and post was not significant (*P* = 0.384). No significant differences were observed between placebo and caffeine at pre (*P* = 0.433), post (*P* = 0.747), or post-YYIR1 (*P* = 0.087). The means and SDs of the cardiovascular variables across different time points are presented in Table 2 and Figure 2, whereas the pairwise comparisons [mean differences (MDs), 95% confidence intervals (CIs), and P values] are detailed in Table 3.

**TABLE 2:** Means and SD of cardiovascular variables (*n* = 12)

Variables	Time	Mean	SD
HR (bpm)	Preplacebo	78.66	8.02
	Postplacebo	90.91	5.88
	Post-YYIR1 placebo	112.33	18.10
	Precaffeine	82.75	8.85
	Postcaffeine	91.75	10.34
	Post-YYIR1 caffeine	123.75	8.94
SBP (mmHg)	Preplacebo	124.83	10.39
	Postplacebo	118.66	12.24
	Post-YYIR1 placebo	126.00	12.51
	Precaffeine	122.13	5.29
	Postcaffeine	118.75	9.79
	Post-YYIR1 caffeine	127.75	13.99
DBP (mmHg)	Preplacebo	80.75	6.86
	Postplacebo	75.66	7.79
	Post-YYIR1 placebo	76.60	9.62
	Precaffeine	75.50	5.64
	Postcaffeine	79.41	4.96
	Post-YYIR1 caffeine	77.41	5.72
RPP (bpm·mmHg/100)	Preplacebo	98.01	11.55
	Postplacebo	107.91	13.60
	Post-YYIR1 placebo	141.71	27.45
	Precaffeine	101.47	13.66
	Postcaffeine	109.00	15.48
	Post-YYIR1 caffeine	158.31	22.81

Abbreviations: BP, blood pressure; DBP, diastolic blood pressure; HR, heart rate; RPP, rate pressure product; SBP, systolic blood pressure; YYIR1, Yo-Yo intermittent recovery test level 1.

**FIGURE 2:**
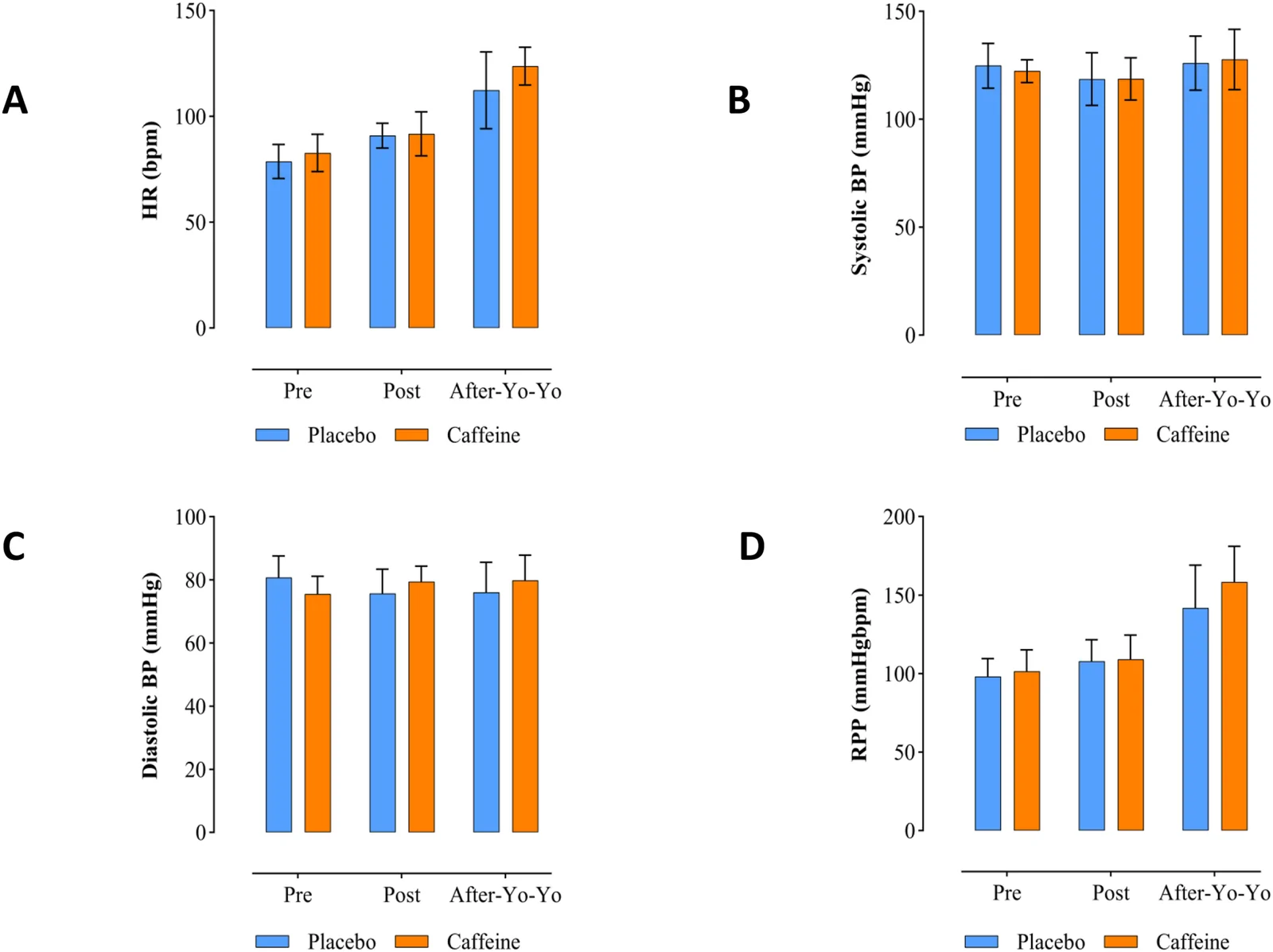
Mean and SD of (A) heart rate (HR), (B) systolic blood pressure (BP), (C) diastolic BP, and (D) rate pressure product (RPP).

**TABLE 3:** Bonferroni pairwise comparisons with MD, 95% CI and *P* values of cardiovascular variables (*n* = 12)

Variables	Comparison	MD	95% CI	*P* value
HR (bpm)	Placebo: pre vs. post	11.92	4.30, 19.53	0.002
	Placebo: pre vs. post-YYIR1	32.42	18.07, 46.76	<0.001
	Placebo: post vs. post-YYIR1	20.50	7.58, 35.29	0.004
	Caffeine: pre vs. post	8.17	−0.27, 16.60	0.064
	Caffeine: pre vs. post-YYIR1	41.67	27.50, 55.84	<0.001
	Caffeine: post vs. post-YYIR1	27.92	15.95, 43.89	<0.001
	Pre: placebo vs. caffeine	−5.75	−13.17, 1.67	0.128
	Post: placebo vs. caffeine	−1.25	−7.93, 5.43	0.796
	Post-YYIR1: placebo vs. caffeine	−11.08	−20.66, −1.51	0.025
SBP (mmHg)	Placebo: pre vs. post	7.58	−0.56, 15.73	0.084
	Placebo: pre vs. post-YYIR1	11.67	−1.54, 24.87	0.129
	Placebo: post vs. post-YYIR1	1.42	−7.96, 10.79	1.000
	Caffeine: pre vs. post	−2.17	−10.02, 5.69	0.636
	Caffeine: pre vs. post-YYIR1	−10.33	−20.79, 0.12	0.054
	Caffeine: post vs. post-YYIR1	−9.08	−18.62, 0.46	0.080
	Pre: placebo vs. caffeine	−2.75	−10.44, 4.94	0.475
	Post: placebo vs. caffeine	0.22	−7.36, 7.80	0.980
	Post-YYIR1: placebo vs. caffeine	3.64	−4.61, 11.89	0.744
DBP (mmHg)	Placebo: pre vs. post	5.08	−1.34, 11.51	0.142
	Placebo: pre vs. post-YYIR1	4.75	−3.10, 12.60	0.348
	Placebo: Post vs. post-YYIR1	−0.33	−6.47, 5.80	1.000
	Caffeine: pre vs. post	−3.92	−9.45, 1.62	0.214
	Caffeine: pre vs. post-YYIR1	−4.33	−11.07, 2.40	0.291
	Caffeine: post vs. post-YYIR1	−0.42	−5.93, 5.10	1.000
	Pre: placebo vs. caffeine	5.25	−0.52, 11.02	0.071
	Post: placebo vs. caffeine	−3.75	−8.37, 0.87	0.101
	Post-YYIR1: placebo vs. caffeine	−3.83	−9.37, 1.71	0.156
RPP (bpm·mmHg/100)	Placebo: pre vs. post	9.90	0.20, 19.59	0.045
	Placebo: pre vs. post-YYIR1	43.70	19.21, 68.20	0.001
	Placebo: post vs. post-YYIR1	33.81	11.80, 55.82	0.004
	Caffeine: pre vs. post	7.53	−5.37, 20.42	0.384
	Caffeine: pre vs. post-YYIR1	56.84	36.01, 77.67	<0.001
	Caffeine: post vs. post-YYIR1	49.31	24.70, 73.93	<0.001
	Pre: placebo vs. caffeine	−3.46	−12.83, 5.91	0.433
	Post: placebo vs. caffeine	−1.09	−8.35, 6.17	0.747
	post-YYIR1: placebo vs. caffeine	−16.60	−36.02, 2.83	0.087

Abbreviations: BP, blood pressure; CI, confidence interval; DBP, diastolic blood pressure; HR, heart rate; MD, mean difference; RPP, rate pressure product; SBP, systolic blood pressure; YYIR1, Yo-Yo intermittent recovery test level 1.

### Functional variables

The dependent *t*-test indicated that the interventions had no significant effect on VO_2max_ (MD = 1.158, *t* = 0.670, *P* = 0.517), CD (MD = 136.67, *t* = 0.666, *P* = 0.519), and LSPT time (MD =-2.095, *t* = −1.898, *P* = 0.084).

Although these differences did not reach statistical significance, several variables demonstrated numerical trends that may be of practical relevance. Compared with the placebo condition, caffeine ingestion resulted in a slightly greater ${\text{VO}}_{2\;\max}$ (+2.2%; 54.35 compared with 53.20 $\text{mL}\cdot {\text{kg}}^{-1}\cdot \min^{-1}$; d=0.193, 95% CI: −2.649, 4.965), a greater CD during the YYIR1 (+6.8%; 2136.66 compared with 2000.00 m; d=0.192, 95% CI: −315.46, 588.37), and a shorter LSPT completion time (-3.6%; 55.38 compared with 57.48 s; d=0.548, 95% CI: −4.524, 0.334).

In addition, post-YYIR1 HR (+10.2%; 123.75 compared with 112.33 bpm) and RPP (+11.7%; 158.31 compared with 141.71) were numerically higher after caffeine ingestion. These findings suggest potential practical effects that may warrant further investigation with larger sample sizes. The means, SDs, 95% CIs, and effect sizes of the functional variables are presented in Table 4 and Figure 3.

**TABLE 4:** Means, SDs, 95% CIs, and effect sizes of functional variables (*n* = 12)

Variables	Intervention	Mean	SD	95% CI	Cohen's *d*
VO_2max_ (mL/kg/min)	Placebo	53.20	7.99	−2.649, 4.965	0.193
	Caffeine	54.35	8.07		
CD (m)	Placebo	2000.00	951.17	−315.46, 588.37	0.192
	Caffeine	2136.66	960.82		
LSPT time (s)	Placebo	57.48	12.13	−4.524, 0.334	0.548
	Caffeine	55.38	11.91		

Abbreviations: CD, covered distance; CI, confidence interval; LSPT, Loughborough soccer passing test; VO_2max_, maximum oxygen consumption.

**FIGURE 3:**
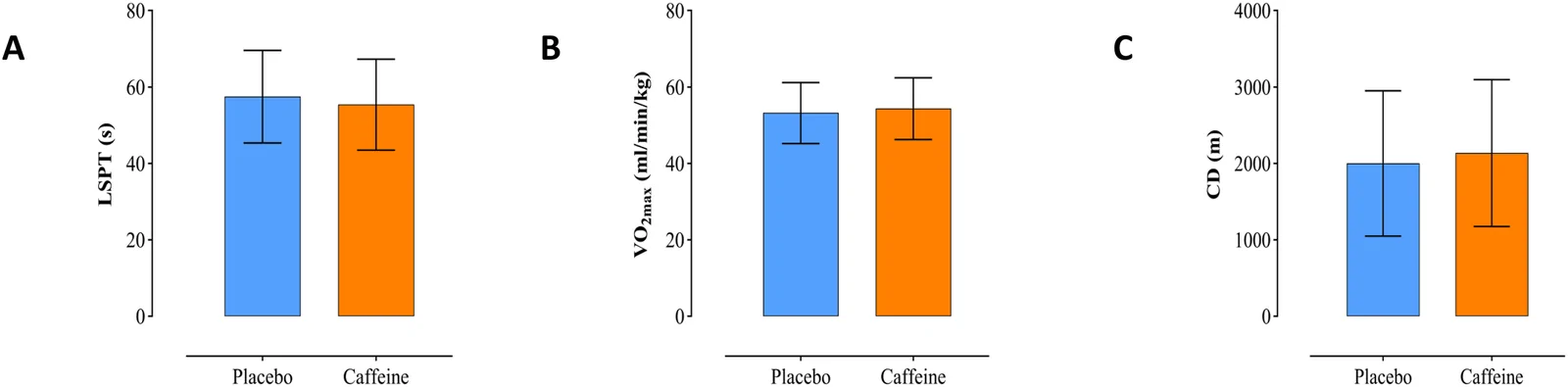
Means and SD of the (A) Loughborough soccer passing test (LSPT), (B) maximum oxygen consumption (VO_2max_), and (C) covered distance (CD).

## Discussion

The main objective of this study was to evaluate the effectiveness of acute caffeine ingestion (3 mg·kg^-1^) on physiological responses and functional variables in adolescent male soccer players.

Regarding cardiovascular responses, acute ingestion of 3 mg·kg^-1^ caffeine produced a significant overall effect on HR, indicating that HR was generally higher during the caffeine trial than during the placebo condition. However, the absence of a significant condition × time interaction suggests that the temporal pattern of HR changes during the experimental protocol was similar under both conditions. Pairwise comparisons further demonstrated that HR was significantly greater after completion of the YYIR1 in the caffeine condition than in the placebo condition, whereas no differences were observed before supplementation or 60 min after ingestion. These findings indicate that the stimulatory effect of caffeine became apparent primarily during maximal intermittent exercise rather than at rest.

Unlike previous investigations that primarily evaluated HR during resting or submaximal exercise, the present study assessed cardiovascular responses immediately after a maximal intermittent field test. Under these conditions, caffeine resulted in a significantly greater postexercise HR compared with placebo, suggesting an augmented sympathetic response during exhaustive exercise. This observation agrees with the well-established pharmacological action of caffeine as an adenosine receptor antagonist, which increases sympathetic activation, particularly when endogenous catecholamine concentrations are already elevated during high-intensity exercise. Consequently, although caffeine did not alter resting HR or postsupplementation values, it appears to modestly augment cardiovascular activation during maximal intermittent exercise in adolescent soccer players.

Unlike HR, SBP was not significantly affected by caffeine supplementation. Although a significant main effect of time was observed, neither the overall effect of condition nor the condition × time interaction reached statistical significance. These findings indicate that SBP changed throughout the experimental protocol regardless of supplementation, suggesting that the physiological demands imposed by the exercise protocol, rather than caffeine ingestion itself, were primarily responsible for the observed fluctuations.

DBP demonstrated a different response pattern. Although neither the main effect of condition nor the main effect of time was statistically significant, a significant condition × time interaction was observed. This finding indicates that the temporal pattern of DBP differed between caffeine and placebo conditions. Nevertheless, subsequent Bonferroni-adjusted pairwise comparisons failed to identify statistically significant differences either between conditions at individual time points or among time points within each condition.

Our findings of nonsignificant updates in SBP and DBP immediately postexercise are consistent with previous findings reporting no difference between the trials for SBP and DBP concentrations [20]. Although higher dosages or multicomponent formulations frequently induce transient increases in resting BP, low doses often show no significant clinical impact, which closely aligns with our outcomes. Collectively, these disparate outcomes across the literature highlight that caffeine’s influence on cardiovascular responses remains context-dependent, largely owing to variations in dosing protocols, formulation types, exercise intensities, and individual habituation status.

In addition, it is critical to acknowledge that BP measurements obtained immediately after exhaustive exercise (e.g., the YYIR1) can exhibit considerable intraindividual variability because of rapidly evolving postexercise hemodynamic responses, including autonomic recovery and dynamic changes in peripheral vascular resistance. Although these cardiovascular fluctuations are inherent, we implemented a standardized immediate auscultation protocol at the left brachial artery, performed by the same examiner, to improve measurement reliability and minimize temporal confounding across conditions.

RPP, an established noninvasive surrogate marker of myocardial oxygen demand and cardiac workload, demonstrated a significant main effect of time but no significant effect of caffeine supplementation or condition × time interaction. These findings indicate that myocardial workload increased progressively throughout the experimental protocol as exercise intensity increased, irrespective of caffeine ingestion. Interestingly, although HR was significantly greater after caffeine ingestion after the YYIR1, this increase was not accompanied by a corresponding increase in SBP. Consequently, the combined myocardial workload represented by RPP did not significantly differ between conditions. This finding suggests that the modest chronotropic effect induced by caffeine was insufficient to substantially increase overall cardiac oxygen demand during maximal exercise. Acute caffeine supplementation did not significantly improve CD during the YYIR1, estimated VO_2max_, or LSPT performance compared with placebo. Nevertheless, all 3 functional outcomes showed numerical improvements after caffeine ingestion. Specifically, CD increased by ∼137 m (6.8%), estimated VO_2max_ increased by ∼2.2%, and LSPT completion time improved by ∼2.1 s (3.6%) under the caffeine condition. Although these improvements failed to reach statistical significance, they may still possess practical relevance in soccer, where even small enhancements in intermittent running capacity and technical execution can influence competitive performance. The relatively small sample size and interindividual variability may have limited the statistical detection of this potential effect.

The discrepancies between our null statistical findings and the positive ergogenic effects reported in previous literature can be attributed to distinct methodological and sample differences. Although studies by Foskett et al. [21] observed significant performance enhancements, they utilized a substantially higher dose of caffeine (6 mg·kg^-1^) and evaluated adult, elite cohorts. In contrast, our study evaluated a low dose (3 mg·kg^-1^) in adolescent players (16.5 ± 0.52 y). From a methodological standpoint, younger athletes possess developing neuromuscular and metabolic systems, which may respond differently to adenosine receptor antagonism compared with fully mature adult players. Furthermore, higher dosages (e.g., 6 mg·kg^-1^) recruit broader peripheral mechanisms, such as enhanced calcium release in skeletal muscle, whereas a low dose primarily acts on the central nervous system. Therefore, the lower dosage combined with the unique physiological profile of adolescent soccer players likely explains why the positive ergogenic outcomes seen in adult protocols did not achieve statistical significance in this study.

Ultimately, several overlapping factors may account for the present null findings. First, the statistical power was constrained because the sample size calculation assumed a very small effect size (d=0.2) an assumption that is arguably unrealistic for acute ergogenic interventions. As a result, the nonsignificant (null) findings should not be interpreted as a definitive lack of efficacy but rather as a reflection of insufficient sensitivity to detect small-to-moderate functional improvements.

Second, substantial interindividual variability was observed in both physiological and performance outcomes, which often washes out group-level statistical significance. This variability is tightly linked to individual caffeine sensitivity and genetic variations (such as the Cytochrome P450 Family 1 Subfamily A Member 2 [CYP1A2] and Adenosine A2a Receptor [ADORA2A] polymorphisms), which dictate whether an athlete is a fast or slow metabolizer. Although our participants were classified as low habitual caffeine consumers (64 ± 15 mg·d^-1^), variations in baseline receptor density and daily dietary habits might still affect individual responsiveness to a low 3 mg·kg^-1^ dose. This study had several limitations that should be acknowledged. Firstly, we did not measure plasma caffeine levels after ingesting caffeine capsules. This could have provided valuable insights into caffeine absorption rates and their relationship with performance outcomes. In addition, the study exclusively involved male soccer players, limiting the generalizability of the findings to other populations, including female athletes and individuals with different training backgrounds.

A key limitation is that the calculated sample size assumed a very small effect size (d=0.2), which is largely unrealistic for acute performance interventions in sports science. Consequently, although our sample size (n=12) matched this initial calculation, it was likely underpowered to detect moderate yet practically meaningful performance enhancements, directly impacting the interpretation of our null results. However, the relatively high variability observed in some outcomes (such as postexercise BP, HR, and RPP), especially given this sample size, could have limited the statistical power to detect significant differences despite the observed numerical trends. Therefore, this high interindividual variability should be acknowledged as a factor influencing the statistical results. It is suggested that, to have more confidence in the results, a larger number of participants should be used in future studies to better account for this variability.

In conclusion, acute ingestion of $3\;\text{mg}\cdot {\text{kg}}^{-1}$ caffeine resulted in modest physiological and functional responses in adolescent male soccer players. Although statistical significance was not reached for soccer-specific performance measures (YYIR1 running distance, estimated VO_2max_, or LSPT performance), consistent numerical improvements were observed across all functional outcomes. Given the study’s limited sample size and statistical power, these preliminary findings should be interpreted with caution rather than as definitive proof of a lack of efficacy.

In practice, coaches and athletes should recognize that although low-dose caffeine may not guarantee acute performance enhancements, individual responses can vary.

These results are limited by the small sample size, single low dose, and interindividual variability. Future studies utilizing larger sample sizes, higher/varied dosages, and stratification by caffeine habituation and genetic profiles (e.g., *CYP1A2* and *ADORA2A*) are needed to determine optimal individualized supplementation strategies.

## Declaration of generative AI and AI-assisted technologies in the writing process

The authors declare that no generative AI or AI-assisted technologies were used in the writing process or scientific content of this manuscript.

## Author contributions

The authors’ responsibilities were as follows – MMA: collected the data and wrote the initial draft; MMA, MS: designed the research, analyzed the data, interpreted the data, critically revised the manuscript, took primary responsibility for the final content, and read and approved the final manuscript.

## Data availability

Data described in the manuscript, codebook, and analytic code will be made available upon request, pending approval from the corresponding author and the relevant institutional ethics committee.

## Funding

The authors reported no funding received for this study.

## Conflict of interest

The authors report no conflicts of interest.
